# The Function of Pre-mRNA Alternative Splicing in Mammal Spermatogenesis

**DOI:** 10.7150/ijbs.34422

**Published:** 2020-01-01

**Authors:** Huibin Song, Ling Wang, Dake Chen, Fenge Li

**Affiliations:** 1 Key Lab of Swine Genetics and Breeding of Ministry of Agriculture and Rural Affairs & Key Laboratory of Agricultural Animal Genetics, Breeding and Reproduction of Ministry of Education, Huazhong Agricultural University, Wuhan 430070, PR China; 2 The Cooperative Innovation Center for Sustainable Pig Production, Wuhan 430070, PR China

**Keywords:** Alternative splicing, Mammal spermatogenesis, Splicing factors, Male infertility

## Abstract

Alternative pre-mRNA splicing plays important roles in co-transcriptional and post-transcriptional regulation of gene expression functioned during many developmental processes, such as spermatogenesis. The studies focusing on alternative splicing on spermatogenesis supported the notion that the development of testis is regulated by a higher level of alternative splicing than other tissues. Here, we aim to review the mechanisms underlying alternative splicing, particularly the splicing variants functioned in the process of spermatogenesis and the male infertility. There are five points regarding the alternative splicing including ⅰ) a brief introduction of alternative pre-mRNA splicing; ⅱ) the alternative splicing events in spermatogenesis-associated genes enriched in different stages of spermatogenesis; ⅲ) the mechanisms of alternative splicing regulation, such as splicing factors and m^6^A demethylation; ⅳ) the splice site recognition and alternative splicing, including the production and degradation of abnormal transcripts caused by gene variations and nonsense-mediated mRNA decay, respectively; ⅴ) abnormal alternative splicing correlated with male infertility. Taking together, this review highlights the impacts of alternative splicing and splicing variants in mammal spermatogenesis and provides new insights of the potential application of the alternative splicing into the therapy of male infertility.

## Introduction

Alternative splicing is one of the most popular co-transcriptional and post-transcriptional regulatory mechanisms that result in a large number of mRNA and protein isoforms from a single gene, and the protein isoforms always show different or mutually antagonistic functional and structural characteristics 1, 2. Recent high-throughput analyses have shown the abundance of alternative splicing events reaching > 95-100% in human genes and 63% in mouse genes 3, 4, and identified several modes of alternative transcript events, including exon-skipping (ES), intron-retention (IR), alternative 5' splice site (A5SS), alternative 3' splice site (A3SS), alternative first exon (AFE), alternative last exon (ALE) and mutually exclusive exon (MXE) 5. The exon skipping events are accumulated in the brain and testis, suggesting a tissue-specific nature of alternative splicing 6, 7.

The process of spermatogenesis occurs in the seminiferous tubules of testis. A_single_ spermatogonia self-renew and form spermatogonial stem cells to ensure the maintenance of the stem cell pool, while A_paired_ spermatogonia usually differentiate into two B spermatogonia 8, 9. B spermatogonia come into the process of mitosis and proliferate into primary spermatocytes. Subsequently, primary spermatocytes divide twice during meiosis and form haploid round spermatids. In this phase, genetic material recombines mutually and the cells constantly split twice 10. Finally, round spermatids go through the process of spermiogenesis to form spermatozoa. In this phase, the morphological changes have taken place in spermatids, including the condensation of nucleus, the formation of acrosome and tail, the elimination of residual body and the transient appearance of manchette 11. The complicated physiological process requires specific genes to perform certain regulatory functions. Recently, high-throughput sequencing has revealed the importance of alternative splicing in the testis proteome diversification and spermatogenesis 5, 12-15. In this review, we will provide the functions of alternative splicing in spermatogenesis, clarify the mechanisms of alternative splicing in spermatogenesis, and explain the potential correlations between alternative splicing and male infertility.

## Alternative splicing of key genes involved in spermatogenesis

Spermatogenesis is a highly complex process that initiates shortly after birth and continues until old age 16. The alternative splicing events are particularly prevalent in the testis, just less popular than in the brain. Spermatogenesis is an extremely complex and coordinated process, and the various genes are dynamically expressed in each type of spermatogenic cells. For better understanding the precision of gene regulation in spermatogenesis, we offer a series of alternative splicing events in the process of spermatogenesis.

GO analysis had identified 242 differently expressed genes enriched in spermatogenesis (GO: 0007283) in the testes from Large White pig at the age of 60 days (60-d) and 180 days (180-d) by RNA-seq 5. Several genes was identified with alternative splicing to generate different transcripts encoding numerous protein isoforms, such as *Spata3* 17, *Spata19* 18, *Crem* 19, 20, *Dazl* 21, *Hsf1* 22, *Acrbp* 23, *Ybx3* 24. Two members of SPATA family, *Spata3*, *Spata19* can generate two splicing variants by exon skipping or alternative 3' splice site, respectively. *Crem*, a transcription factor regulated by cAMP controls the developmental progression of germ cells, and has ten splicing variants via several modes of alternative splicing. In postnatal testis developmental stages, the *Dazl-Δ8* isoform is constantly expressed, along with *Dazl-FL* isoform. The information about spermatogenesis-enriched genes and their alternative splicing events are summarized in Table 1.

**Figure 1:**
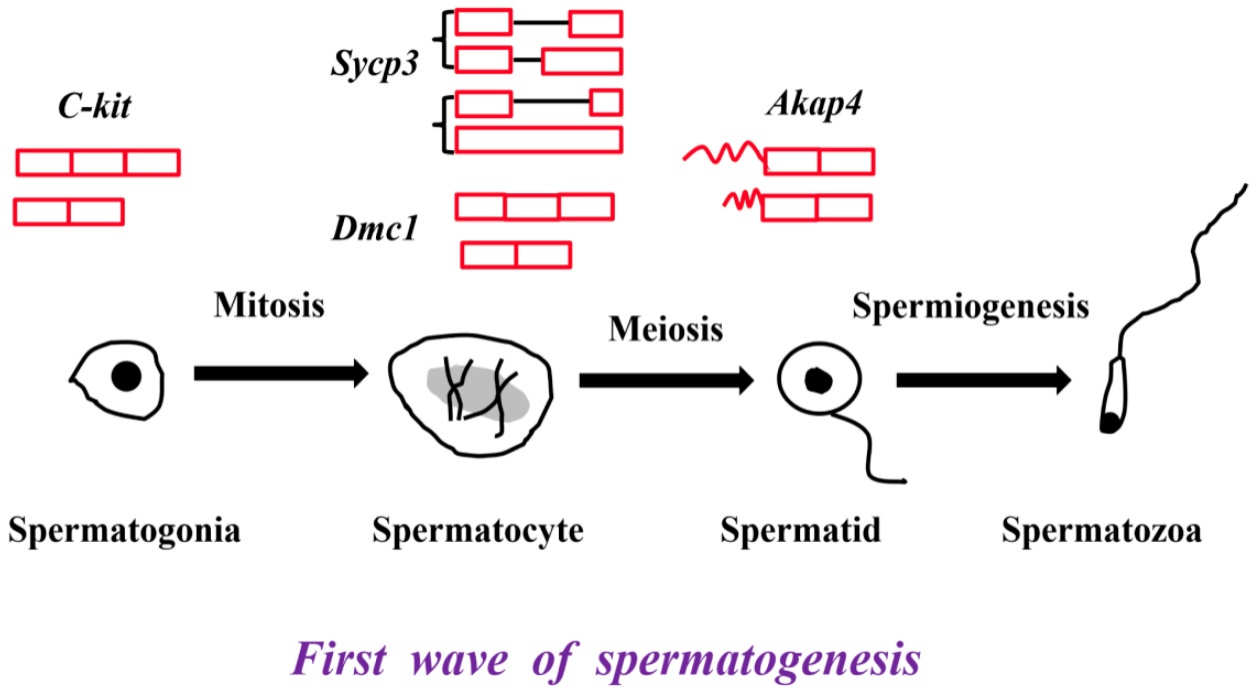
The critical genes and their alternative splicing transcripts in the first wave of spermatogenesis. *C-kit* is the marker of differentiated spermatogonial cells. Two transcripts, full length transcript and the exon skipped transcript were detected in mouse testis 26, 27. *Sycp3* and *Dmc1* are the markers of spermatocytes. The A3SS transcript and retained intron transcript of *Sycp3* were identified in the testes from 60-d and 180-d Large White pigs 5. The exon skipped Dmc1-d transcript was expressed in both male and female germ cells 31. *Akap4* is the marker of spermatids. Two *Akap4* alternative splicing variants - *Akap82* and *Fsc1* were different in their 5' UTR 33.

Many germ cell-specific transcripts are developmentally regulated and stage specific 25. C-kit, proto-oncogene receptor tyrosine kinase, plays an indispensable role in the differentiation of spermatogonial cells 26. A truncated form *tr-kit* is specifically expressed in spermatids and spermatozoa, and acts as a putative sperm factor required for triggering activation of mouse eggs at fertilization 26, 27. Synaptonemal complex protein 3 (Sycp3), specifically localized in spermatocytes, is necessary for male meiosis and spermatogenesis 28, 29. In our previous study, an A3SS transcript and a retained intron transcript of *Sycp3* gene were identified in the testes from 60-d and 180-d Large White pigs 5. Disrupted meiotic cDNA1 (Dmc1) is required for double-strand break repair and plays a pivotal role during meiotic homologous recombination 30. An exon skipped transcript *Dmc1-d* is expressed in both male and female germ cells, which indicates a novel role in meiosis 31. A-kinase anchoring protein 4 (Akap4) is transcribed only in the post-meiotic phase of spermatogenesis and *Akap4* knockout mice exhibit defects in sperm flagellum and motility 32. *Akap82* and *Fsc1* belonging to Akap4 alternative splicing variants are different in 5' UTR, and therefore encode the identical proteins, but *Akap82* transcript is more abundantly expressed in spermatids than *Fsc1* transcript (Figure 1) 33.

Sperm-associated antigen families are the proteins that highly expressed in the testis and are essential for motile cilia and flagella 34. Previous studies have indicated that alternative splicing of sperm-associated antigen genes play important roles in spermatogenesis 5, 35-40. Three isoforms of human Spag11b including Spag11b-a, Spag11b-d and Spag11b-g were different in their 3D fold structure 35. Spag11b-d isoform could interact with tryptase alpha/beta 1 (Tpsab1), tetraspanin 7 (Tspan7), and attractin (Atrn), and then played major roles in immunity and fertility 36. Two *Spag11* transcripts including *Spag11c* and the exon-skipped transcript *Spag11t* were detected in rat 37. Spag11c was expressed in epididymis and testis, while Spag11t was only confined to the caput region in the epididymis and was absent from testis and seminal vesicle 37. Spag16 is the homologous to *Chlamydomonas reinhardtii* PF20 and is associated with the axonemal central apparatus 38. In human testis, the long Spag16 isoform was localized to the central microtubule of the sperm, whereas the short isoform was located around the nucleus of spermatogenic cells in the late stage of meiosis 39, 40. RNA-sequencing of immature and mature porcine testes identified several alternative splicing events of *Spag6* gene 5. Overall, the alternative splicing events of sperm-associated antigen family are spatio-temporal expressed, which illustrate the crucial role in spermatogenesis.

## The mechanisms of alternative splicing regulation

Eukaryotic genes are composed of short exons and long introns. A majority of pre-mRNAs exist constitutive splicing and alternative splicing. Both types of splicing follow the “GT-AG” rule and are regulated by *cis*-acting splicing regulatory elements (SRE) and *trans*-acting splicing factors. The SREs consist of exonic splicing enhancers and silencers (ESE and ESS), intronic splicing enhancers and silencers (ISE and ISS) 41, 42. The *trans*-acting factors are some RNA binding proteins such as the serine/arginine-rich (SR) proteins and heterogeneous nuclear ribonucleoprotein (hnRNP) family. SR proteins usually promote pre-mRNA splicing by binding ESE or ISE sequences via the N-terminal RNA recognition motifs (RRMs) or by interacting with other proteins via the C-terminal arginine-and serine-rich domain (RS), while hnRNP family usually inhibits pre-mRNA splicing by binding ESS or ISS sequences via their RRMs 43. Generally, SR and hnRNP proteins have the opposite function in RNA splicing. However, the recent study in *Drosophila* indicates that SR and hnRNP proteins tend to act coordinately but not antagonistically 44.

RNA binding proteins can act as splicing enhancers or repressors, depending on which region of a skipped exon they bind to 45, 46. Recent researches have revealed the critical role of RNA binding proteins in mammal spermatogenesis 47-50. The polypyrimidine tract binding protein 2 (Ptbp2) is a strong regulator of alternative splicing 48, 52, 53. In mammal spermatogenesis, *Ptbp2* binds 3'SS of the cassette exons in critical genes, inhibits the exon skipping and then regulates alternative splicing in a stage-specific manner 48, 51. Furthermore, numerous mis-spliced isoforms in those genes that are essential for Sertoli cell cytoskeleton and germ cell-Sertoli cell crosstalk appeared in* Ptbp2*-deficient mouse testis 48. Dazap1 is a ubiquitous hnRNP protein that is expressed most abundantly in the testis and knock down of *Dazap1* in 293T cells exhibits alternative splicing changes in those genes involved in cell cycle, DNA replication, transcriptional control and metabolism 49. Another study revealed that a missense mutation (R263P) in the second RRM of RNA binding motif protein 5 (Rbm5) affected pre-mRNA splicing, produced aberrantly spliced transcripts and displayed spermatid differentiation arrest, azoospermia and male sterility 50.

*N*^6^-methyladenosine (m^6^A) is a kind of the most abundant modifications in messenger RNAs and plays a pivotal role in regulating alternative splicing and RNA degradation 54. RNA m^6^A methylation is a dynamic and reversible modification that mediated by m^6^A “writers”, “erasers” and “readers” 55-61. m^6^A “writers” mainly consist of methyltransferases like 3 and 14 (Mettl3 and Mettl14), WT1 associated protein (Wtap) and vir like m6A methyltransferase associated (Virma) 55-57. While alpha-ketoglutarate dependent dioxygenase (Fto) and alkB homolog 5 (Alkbh5) as demethylases (m^6^A “erasers”) can reverse the m^6^A methylation 58, 59. And currently, m^6^A “readers” have been found including the binding proteins family such as heterogeneous nuclear ribonucleoprotein family and YTH N6-methyladenosine RNA binding proteins 60, 61.

**Figure 2:**
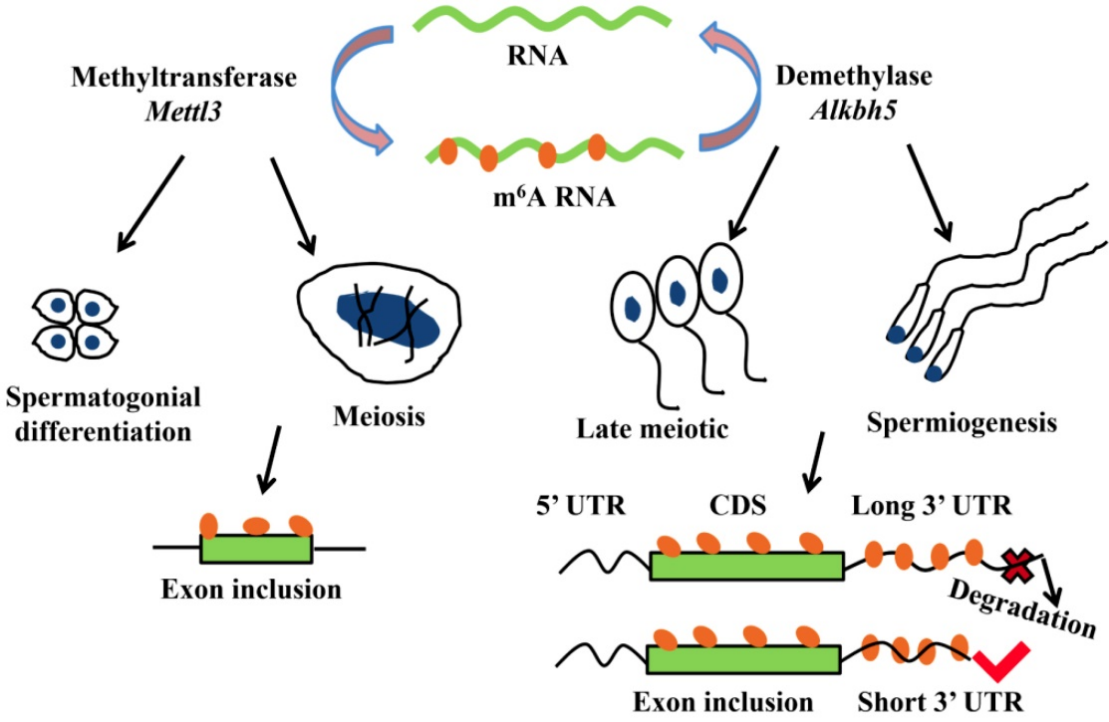
m^6^A modification plays a critical role in mRNA alternative splicing and stability in mammal spermatogenesis. m^6^A methyltransferase *Mettl3* is essential for spermatogonial differentiation and meiosis during mouse spermatogenesis. m^6^A modification that mediated by *Mettl3* ensures exons containing m^6^A sites have the correct exon inclusion levels 63. m^6^A demethylase Alkbh5 is required for the late meiotic and spermiogenesis during spermatogenesis 65. m^6^A tends to mark the 3'-UTRs of longer mRNAs that are destined to be degraded and *Alkbh5* controls correct splicing of exon and 3'-UTR which have m^6^A sites 66, 67.

More and more researches show m^6^A modification plays a critical role in mRNA alternative splicing and stability in mammal spermatogenesis 59, 62. m^6^A methyltransferase *Mettl3* knockout mouse shows the defects in spermatogonial differentiation and meiosis, with a lower exon inclusion level in those transcripts containing m^6^A and alternative splicing in several critical genes involved in spermatogenesis including *Dazl*, *Sohlh1*, *Nasp* and *Cdk11b* 63. During late spermiogenesis, the increased production of transcripts with shorter 3'-UTRs allows for an efficient translation and a quick mRNA/protein turnover 64. m^6^A demethylase Alkbh5 is required for the late meiotic and haploid phases of spermatogenesis 65. In spermatocytes and round spermatids, *Alkbh5* tends to mark the coding sequences and 3'-UTRs of longer mRNAs that are destined to be degraded and controls correct splicing of long 3'-UTR transcripts (Figure 2) 66, 67.

## The splice site recognition and alternative splicing

**Figure 3:**
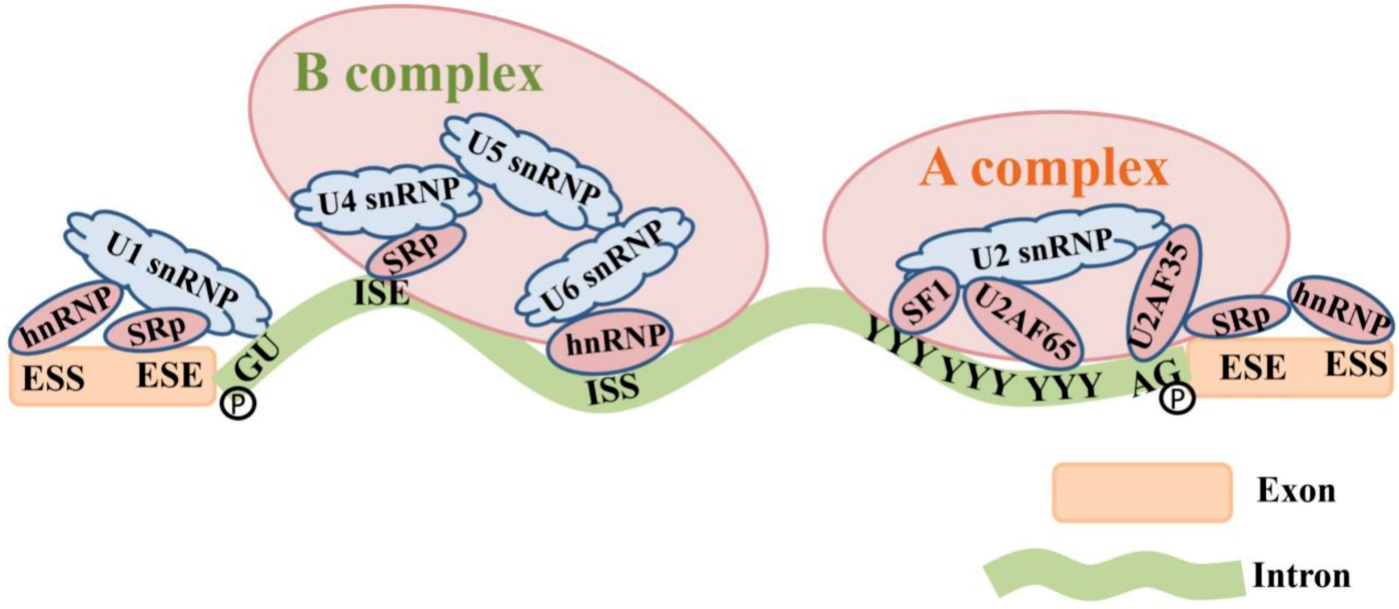
The composition of the spliceosome complex and the pre-mRNA splicing process. U1 snRNP binds to the 5' GU. U2 snRNP and SF1 bind to the branch site under the assistance of the mRNA splicing factor U2 associated factor (U2AF65 and U2AF35) and form the splicing complex precursor (A complex) 69. U4-U5-U6 snRNP trimer forms the splicing complex (B complex) through interactions between RNA-RNA (SR proteins) that bind to ISE and RNA-proteins (hnRNP) that bind to ISS. The 5' end and 3' end of the intron are cleaved from the upstream and downstream exons by a 2', 5'-phosphodiester linkage, and therefore exons are connected together.

The pre-mRNA splicing is originated by spliceosomes that bind to sequences located at the 5' and 3' ends of introns 68. Spliceosome assembly comprises five types of small nuclear ribonucleoproteins (snRNPs). U1 snRNP binds to the 5' GU. U2 snRNP and splicing factor 1 (SF1) bind to the branch site under the assistance of the mRNA splicing factor U2 associated factor (U2AF) and form the splicing complex precursor (A complex) 69. U4-U5-U6 snRNP trimer forms the splicing complex (B complex) through interactions between RNA-RNA (SR protein) and RNA-protein (hnRNP) 1. Subsequently, 5' end of the intron is cleaved from the upstream exon and joined to the branch site by a 2', 5'-phosphodiester linkage. The 3' end of the intron is cleaved from the downstream exon, and the two exons are joined by a phosphodiester bond 41. The intron is then released in lariat form and degraded 70 (Figure 3).

Alternative splicing always comes with the emergence of the improper splice site recognition, which is always influenced by genetic variants or abnormal expression of splicing factors 41. For example, an exonic SNP (c.2851G>T) in sperm flagellar 2 (Spef2) is associated with semen deformity rate and post-thaw cryopreserved sperm motility in Holstein bulls, potentially leads to the production of *Spef2-SV3* transcript which is only detected in testis and epididymis 71. Inner centromere protein (Incenp) is involved in cell division and sister chromatid separation as the main member of chromosomal passenger protein complex and a mutation (g.19970A>G) in its intron 11 results in exon 12 skipping and creates several novel binding sites for the splicing factors SRSF1, SRSF5, and SRSF6 72. The SR proteins, such as SRp38 and 9G8, are highly expressed in germ cells 73, 74. The SNPs in these SR proteins are significantly associated with the risk of non-obstructive azoospermia in Chinese men, which provides more evidence for the role of splicing activity in human spermatogenesis 75. A SNP in exonuclease 1 (Exo1) intron 8 potentially results in the production of truncated forms-*tr1-Exo1* and *tr2-Exo1*, which partly explains metaphase-specific apoptosis in MRL/MpJ mice 76. Zrsr1 is a U2AF35-like splicing factor which recognizes the 3' splice site during spliceosome assembly, and the *Zrsr1* mutant mice containing truncating mutations within its RRM exhibits the abundant intron retention events in genes associated with spermatogenesis, and germ cell apoptosis, azoospermia and male sterility 77. The conditional deficiency of another pre-mRNA splicing factor breast cancer amplified sequence 2 (Bcas2) in male germ cells results in male infertility and aberrant splicing of *Dazl*, *Ehmt2* and *Hmga1* genes 78.

**Figure 4:**
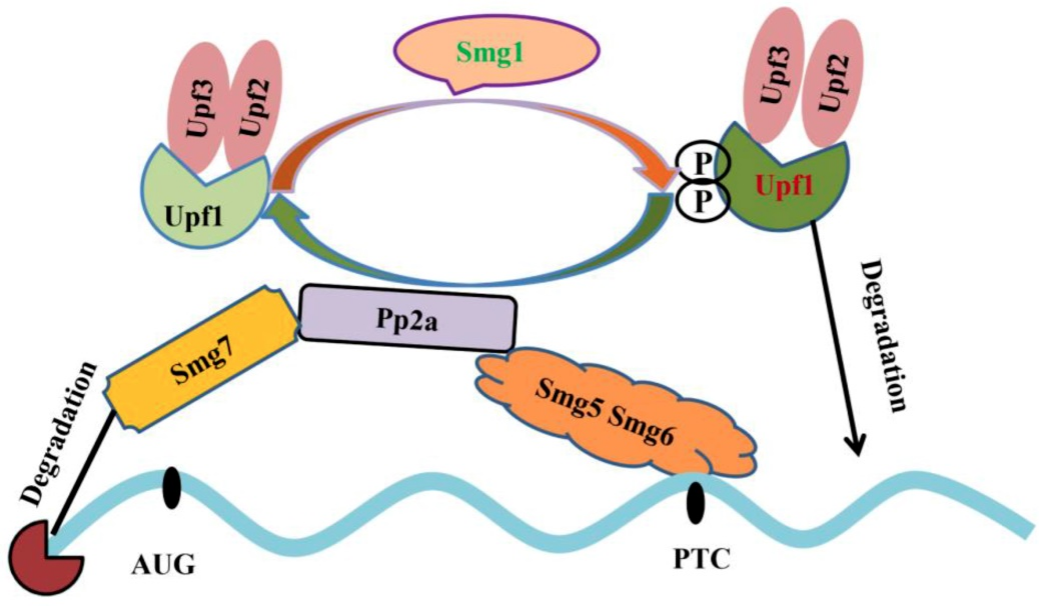
Mechanistic model underlying nonsense-mediated mRNA decay. Upf1 interacts with Upf2, Upf3 and then undergoes Smg1-mediated phosphorylation. Pp2a interacts with Smg5, Smg6 and Smg7 and promotes Upf1 de-phosphorylation. Smg5 and Smg6 identify PTC; Smg7 degrade mRNA 5'cap structure and phosphorylated-Upf1 degrades mRNAs 81-84.

Alternative splicing coupled to nonsense-mediated mRNA decay (NMD) is an efficient strategy to regulate gene expression. The main feature of NMD is that the last exon generates a premature termination codon (PTC) producing a transcript with a C-terminally truncated polypeptide. NMD pathway is a post-transcriptional RNA surveillance mechanism that rapidly degrades the toxic truncated protein, preventing it from damaging cells 79, 80. Up-frameshift mutant (UPF) and suppressor with morphological effect on genitalia (SMG) family are important factors in NMD pathway. Upf1 recognizes abnormal translation termination and then interacts with Upf2 and Upf3 81. Upf1 phosphorylation is mediated by Smg1. Protein phosphatase 2A, Pp2a interacts with Smg5, Smg6 and Smg7, and then promotes Upf1 de-phosphorylation. Smg5 and Smg6 identify PTCs by their special domains and degrade mRNAs (Figure 4) 82-84. The mRNAs that contain PTCs or long 3' UTRs could be the potential substrates of NMD 85. Emerging evidence provide an indispensable role of NMD in spermatogenesis. Inactivation of *Upf2*, a component of the chromatoid body, causes azoospermia and male sterility. In *Upf2*-null mouse spermatocytes and round spermatids, the long 3' UTR transcripts from ubiquitously expressed genes are abundantly accumulated and trigger nonsense-mediated mRNA decay 86. Tudor domain containing 6 (Tdrd6) is essential for Upf1-Upf2 interaction. The long 3' UTR-stimulated NMD is impaired in *Tdrd6^-/-^* spermatids by interfering with Upf1-mRNA binding, thus perturbing mRNA processing 87.

## Alternative splicing and male infertility

Spermatogenesis is a continuous hormone-dependent cell proliferation and differentiation process. Follicle-stimulating hormone (FSH) and luteotropic hormone (LH) can induce the secretion of testosterone that is important for spermatogenesis 88. In addition, FSH and LH are also essential for the initiation and maintenance of normal spermatogenesis 89. Multiple studies have found that alternative splicing of hormone receptor genes influences male infertility 93-96. For example, a homozygous G>A mutation in intron 10-exon 11 boundary of *LHR* gene results in skipping of partial exon 11. The male patient appears delayed puberty, micropenis and oligospermia, and two of his sisters are infertile 90. Several splicing variants in *FSHR* gene are identified in infertile patients, including the exon 9 skipped variant which leads to the removal of two highly conserved cysteine residues at positions 275 and 276 91, 92. Another study reveals several *FSHR* splicing variants lack of exon 2, exon 2 and 5, exon 5 and 6, exon 2, 5 and 6 in mouse testis 93. The exon 5 and 6 skipped transcript produces a mutant receptor that can't bind to FSH and then leads to male infertility.

**Figure 5:**
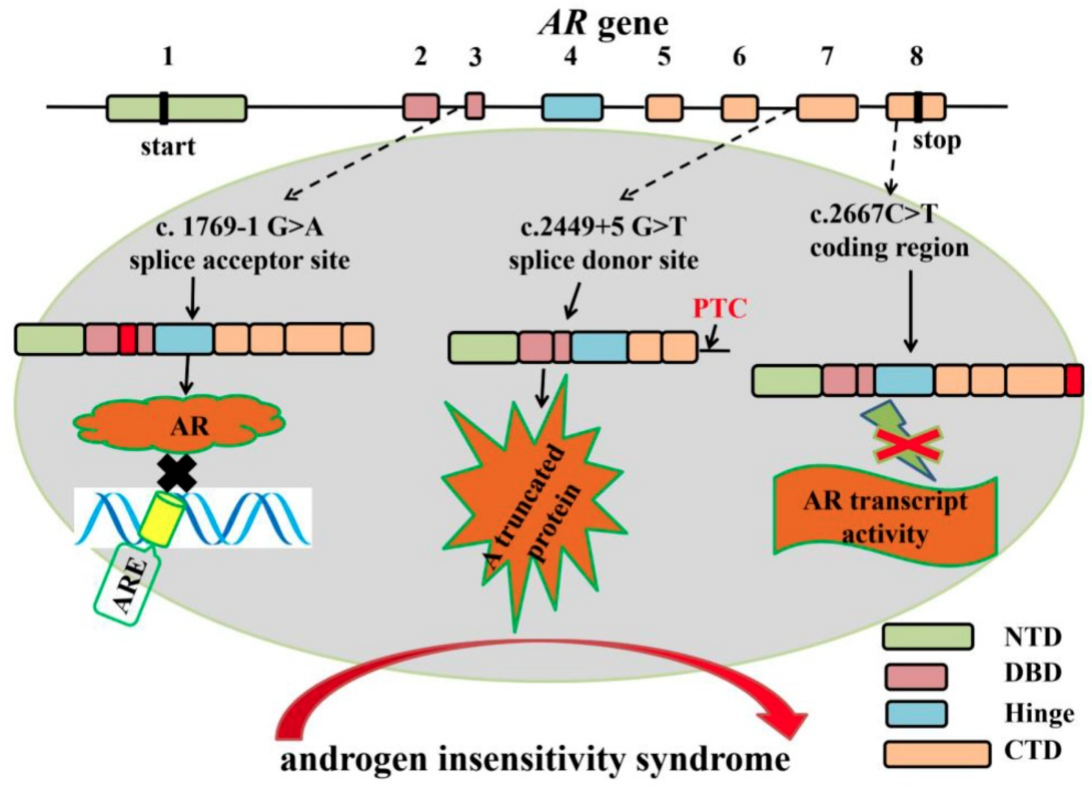
The mutations in *AR* gene splice sites result in aberrant splicing which are closely associated with human androgen insensitivity syndrome. Boxes represent exons of human *AR* gene. Exon 1 encodes N-terminal transactivation domain (NTD); Exon 2 and 3 encode the first and second zinc fingers of DNA-binding domain (DBD); Exon 4 encodes the hinge region; Exon 5-8 encode the COOH-terminal domain (CTD). Several mutations in *AR* gene splice sites result in aberrant splicing in androgen insensitivity syndrome patients. For example, the c.1769-1G>A mutation in intron 2 splice acceptor site results in an insertion of 69 nucleotides. The insertion between the two zinc fingers of the AR DNA binding domain (DBD domain) impairs the specific binding of AR response elements to AR 99, 100. A c.2449+5G>T mutation in intron 6 boundary splice donor site gives rise to a truncated protein that lacks part of C-terminal ligand-binding domain 101. A c2667C>T mutation in exon 8 produces an aberrant splicing variant that leads to partial skipping of exon 8 and a shortened 3'-untranslated region and the androgen-induced transcriptional activity is inhibited 102.

Previous studies showed that Sertoli-cell specific androgen receptor (AR) knockout mouse exhibited spermatogenic arrest and male infertile, suggesting the indispensable role of AR in spermatogenesis 94-97. Human AR protein consists of four functional domains: N-terminal transactivation domain (NTD) encoded by exon 1; DNA-binding domain (DBD) encoded by exon 2 and 3; Hinge domain encoded by exon 4 and ligand-binding domain (LBD) encoded by exon 5-8 98. Several mutations in *AR* gene splice sites are closely associated with androgen insensitivity syndrome 99-102. The c.1769-1G>A mutation in intron 2 splice acceptor site of* AR* gene results in an insertion of 69 nucleotides, which creates an insertion of 23 amino acids 99. The insertion that located between the two zinc fingers of the AR DBD domain impairs the specific contact from AR and its hormone response element 100. A c.2449+5 G>T mutation in intron 6 boundary splice donor site prevents the normal splicing of intron 6 and gives rise to a truncated protein that contains a PTC in retained intron 6. The truncated protein lacks part of C-terminal ligand-binding domain, and the truncated transcript is highly expressed in an 11-year-old girl, which probably explains the partial androgen insensitivity syndrome 101. A synonymous mutation (C>T) in exon 8 is identified in a patient with partial androgen insensitivity syndrome 102. The mutation produces an aberrant splicing variant that leads to partial skipping of exon 8 and a shortened 3'-untranslated region and the androgen-induced transcriptional activity is inhibited (Figure 5). Overall, aberrant alternative splicing of hormone receptor genes is closely associated with spermatogenesis and male infertility.

## Summary and prospects

**Figure 6:**
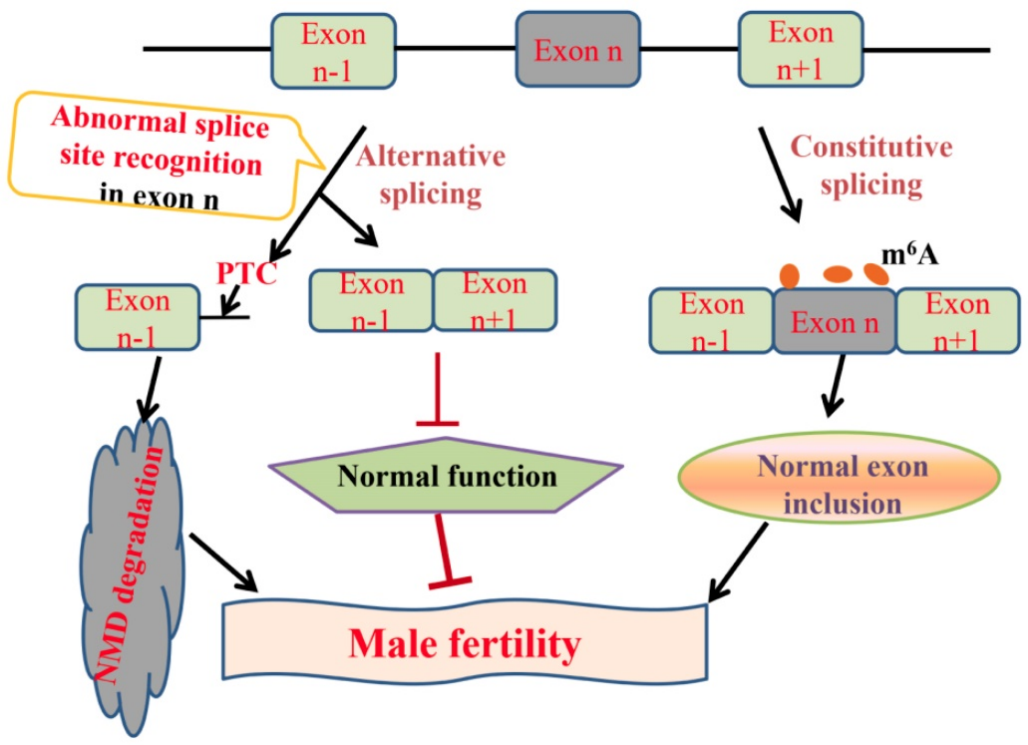
Pre-mRNA splicing in mammal spermatogenesis and male infertility. In normal condition, pre-mRNA undergoes constitutive splicing, removes introns and joins adjacent exons. m^6^A modification maintains correct exon inclusion levels and plays a critical role in mammal spermatogenesis 62-67. Alternative splicing often comes into being along with the emergence of the abnormal splice site recognition. On the one hand, alternative splicing generates abnormal transcripts with a premature termination codon (PTC), which are degraded by nonsense-mediated mRNA decay (NMD) 65, 86, 87. On the other hand, the mutations in splice sites result in the production of exon-skipped transcripts which are closely associated with male infertility 75, 76, 99-102.

We have summarized the current knowledge regarding genes that regulate alternative splicing in spermatogenesis and also investigated the relationships between alternative splicing and male infertility (Figure 6). Spermatogenesis is a strictly regulated process, at both the transcriptional and the post-transcriptional level. Understanding the alternative splicing in spermatogenesis will be helpful to improve the sperm quality and cure male infertility.

It's well known that germ cells differentiate into spermatozoa, and transmit genetic and epigenetic information across generations 103. This review provides a large amount of information about alternative splicing events in germ cells of the genes critical for spermatogenesis. However, testis is a highly complex tissue which contains Leydig cells, Sertoli cells, germ cells and peritubular myoid cells. These cells cooperate with each other and ensure normal spermatogenesis. The Sertoli cells are essential for creating a microenvironment that enables to produce the functional spermatozoa. The communication between Sertoli-Sertoli cell and Sertoli-germ cell constitutes ectoplasmic specialization and blood-testis barrier (BTB) which protect germ cells from immunological attack and provide nutrients for germ cells 104. In addition, Leydig cells that between seminiferous tubules produce growth factors and secrete testosterone. A previous study has shown that LHR plays a key role in testosterone production and eight splicing variants of *LHR* gene are detected in bovine Leydig cells 105. The androgenic stimulation of peritubular myoid cells around seminiferous tubules is also essential for normal germ cell development 106. However, the functions of alternative splicing patterns of critical genes in Sertoli cells, Leydig cells and peritubular myoid cells are worth being explored.

Recently, there have been many researches on non-coding RNAs in spermatogenesis. Circular RNAs (circRNAs), unlike miRNAs and long non-coding RNAs (lncRNAs), are a novel type of non-coding RNAs originated from introns, intergenic regions, untranslated regions and exhibit distinct patterns of alternative back-splicing and alternative splicing 107, 108. The roles of circRNAs in self-renewal and differentiation of spermatogonial stem cells and sperm motility are gradually being studied 109-112. However, the relationships between alternative splicing that involved by circRNAs and spermatogenesis was seldom reported and needed further study.

High-throughput sequencing of short cDNA fragments (RNA-seq) generates tens of thousands alternative splicing events in numerous tissues. Meanwhile, the identification of important alternative splicing transcripts has become an urgent issue. There are two commonly used methods to estimate alternative splicing events at present. One is percent spliced in (PSI), proposed by Wang et al 113. ΔPSI is used to detect differential alternative splicing events in two samples of RNA-seq data. When ΔPSI ≥ 10%, the changes of splicing events in different samples are considered important 114. The other popular method to recognize alternative splicing is alternative splicing detector (ASD), which detects differential alternative splicing exons in different samples of RNA-seq data 115. This software considers the altered junction reads and altered coverage of AS-exons while calculating a *P* value. When *P* < 0.05, the AS event is statistically significant. Currently, both methods have been widely applied in numerous tissues 116-118, but scarcely in the testis. Therefore, the important alternative splicing events in the development of spermatogenesis deserve further excavation and investigation. In general, this study reviews some progresses in alternative splicing of spermatogenesis and also provides new ideas in the therapy of male infertility.

## Figures and Tables

**Table 1:** The information of genes enriched in spermatogenesis and their alternative splicing modes.

Gene name	Species	Ensembl ID	Chromosome location	Variants number	Transcript ID	Modes of alternative splicing	Accession number	Conventional transcript or not	References
Spata3	Mouse	ENSMUSG00000026226	1	2	ENSMUST00000052854	_	NM_027300	Yes	17
					ENSMUST00000152501	Exon skipping	NM_027029	No	
Spata19	Mouse	ENSMUSG00000031991	9	2	ENSMUST00000034473	_	NM_029299	Yes	18
					ENSMUST00000214287	Alternative 3' splicing site	NM_001305058	No	
Crem	Rat 19	ENSMUSG00000063889	18	10	ENSMUST00000025069	_	NM_001271506	Yes	19,20
	Human 20				ENSMUST00000049942	Alternative first exon	NM_001311067	No	
					ENSMUST00000082141	Exon skipping	NM_001271505	No	
					ENSMUST00000122958	Alternative first exon	NM_001110853	No	
					ENSMUST00000124747	Alternative first exon	NM_001311066	No	
					ENSMUST00000130599	Alternative first exon, Exon skipping	NM_001110857	No	
					ENSMUST00000137568	Alternative first exon, Exon skipping	NM_001110850	No	
					ENSMUST00000142690	Alternative last exon	NM_001110851	No	
					ENSMUST00000146265	Exon skipping	NM_001271503	No	
					ENSMUST00000149803	Alternative 3' splicing site	NM_001110852	No	
Dazl	Mouse	ENSMUSG00000010592	17	2	_	_	NM_010021	Yes	21
					ENSMUST00000010736	Exon skipping	NM_001277863	No	
Hsf1	Mouse	ENSMUSG00000022556	15	4	ENSMUST00000072838	_	NM_008296	Yes	22
					ENSMUST00000226872	Alternative 3' splicing site	NM_001331214	No	
					ENSMUST00000227478	Alternative 3' splicing site	NM_001331154	No	
					ENSMUST00000228371	Exon skipping	NM_001331153	No	
Acrbp	Mouse	ENSMUSG00000072770	6	2	ENSMUST00000088294	_	NM_016845	Yes	23
					ENSMUST00000112414	Alternative 3' splicing site	NM_001127340	No	
Ybx3	Mouse	ENSMUSG00000030189	6	2	ENSMUST00000032309	_	NM_139117	Yes	24
					ENSMUST00000087865	Exon skipping	NM_011733	No	

Note:'_'means that there is no information.

## Abbreviations

ES: exon-skipping
IR: intron-retention
A5SS: alternative 5' splice site
A3SS: alternative 3' splice site
AFE: alternative first exon
ALE: alternative last exon
MXE: mutually exclusive exon
SRE: splicing regulatory elements
ESE: exonic splicing enhancers
ESS: exonic splicing silencers
ISE: intronic splicing enhancers
ISS: intronic splicing silencers
SR: serine/arginine-rich
hnRNP: heterogeneous nuclear ribonucleoprotein
m^6^A: *N*^6^-methyladenosine
snRNPs: small nuclear ribonucleoproteins
NMD: nonsense-mediated mRNA decay
PTC: premature termination codon
UPF: Up-frameshift
FSH: follicle-stimulating hormone
LH: luteotropic hormone
AR: androgen receptor
NTD: N-terminal transactivation domain
DBD: DNA-binding domain
LBD: ligand-binding domain
BTB: blood-testis barrier
circRNAs: circular RNAs
PSI: percent spliced in
ASD: alternative splicing detector
